# Home-based carers’ perceptions of health promotion on sexual health communication in Vhembe District

**DOI:** 10.4102/curationis.v38i1.1181

**Published:** 2015-05-05

**Authors:** Dorah U. Ramathuba, Ntsieni S. Mashau, Augustine Tugli

**Affiliations:** 1 Department of Nursing, University of Venda, South Africa; 2 Department of Public Health, University of Venda, South Africa

## Abstract

**Background:**

The introduction of home-based care in rural communities in the 1980s contributed immensely toward the upliftment of the personal and environmental health of communities. Women’s groups provided health promotion skills and health education to communities and made a difference in health-related behaviour change.

**Objective:**

The purpose of the study was to explore and describe the home-based carers’ perception regarding health promotion concerning sexual health communication in Vhembe district, in the context of HIV, amongst communities still rooted in their culture.

**Method:**

A qualitative, explorative and descriptive design was used in order to understand home-based carers’ perceptions regarding health promotion on sexual health communication amongst rural communities which may adversely impact on health promotion practices. The population were home-based organisations in Vhembe. The sample was purposive and randomly selected and data were gathered through semi-structured face-to-face interviews and focus groups which determined data saturation. Open coding was used for analysis of data.

**Results:**

The results indicated that sexual communication was absent in most relationships and was not seen as necessary amongst married couples. Socioeconomic conditions, power inequity and emotional dependence had a negative impact on decision making and sexual communication.

**Conclusion:**

This study, therefore, recommends that educational and outreach efforts should focus on motivating change by improving the knowledge base of home-based carers. Since they are health promoters, they should be able to change the perceptions of the communities toward sexually-transmitted infections and HIV by promoting sexual health communication.

## Introduction

The impact of HIV can be seen through an increase in the number of people living with HIV and AIDS (PLWHA), which has put pressure on hospital staff who are already struggling to cope with their workload. This gave rise to home-based care centres with home-based caregivers (HBCGs) to care for the PLWHA. In developing countries, home-based care (HBC) programmes were first initiated and implemented by churches and other faith-based organisations. In South Africa, HBC programmes were largely non-existent, however care groups started in the late 1980s with the introduction of care groups in the rural villages of Limpopo. Care groups, started as a way of mobilising healthcare practices to promote health, aspects of child care, nutrition, health promotion activities of having toilets, pit toilets and vegetable gardens in their homes and/or households, were some of the health promotion aspects that community nurses worked with in this group of people.

In recent years, HBC centres have become more structured and in most countries they remain in the forefront of service delivery. Some of their activities include delivery of treatment, care and support to PLWHA (Joint United Nations Programme on HIV/AIDS 2006). The South African government has funded the development of home- and community-based care, the training of caregivers and the provision of HBC kits to caregivers. All of these are also included in the strategies to improve HIV care and to reduce the burden experienced by the public sector, as well as caring for PLWHA at home.

Internationally, the term ‘community health worker’ (CHW) and HBCG are used similarly, as their definitions are exhaustive depending on the area/region or context in which they are used; CHWs are called rural health motivators in Swaziland, rural health workers in other countries and female community health promoters or female multipurpose health workers in Nepal. CHWs are selected within communities and undergo some form of basic health information and skills training so that they provide support in the different areas such as tuberculosis and HIV or nutrition. A variety of community health aides are selected, trained and working in their home communities (World Health Organization 1989).

Today, CHWs are widely used as lay counsellors. Lehmann & Sanders (2007) also reported that:

there is a proliferation of community, church and NGO-initiated activities, particularly in countries with high HIV prevalence, which make use of lay personnel for a wide range of prevention, support and care activities. (p. 13)

Vhembe district is not exceptional – most of the lay counsellors started as CHWs. The problem is accounting for and taking responsibility for the training of these CHWs and empowering them; there is no stipulated and regulated training and, depending on the areas where they are providing services, this makes their services inconsistent. The majority of these are women and their roles and activities are enormously diverse, depending on the programme that they were trained for. For this reason, there are specific institutions that train HCWs through South African Qualifications Authority-accredited programmes which have unit standards that should be achieved. It is from this background that the perception of HBCGs was explored in order to determine their role in providing a wide range of different tasks that are preventive, promote in combating the spread of HIV and promote healthy sexual relations.

Contributing factors to the spread of HIV include poverty, social instability and high levels of sexually-transmitted infections (STIs), to name but a few (AIDS Foundation of South Africa 2005).

Many men and women still fail to protect themselves against STIs, including HIV, because they find it difficult, if not impossible, to discuss subjects related to sexuality with their partners. That being said, the desire to maintain a relationship often outweighs health concerns. On the whole, women avoid discussions about safer sex or will talk about AIDS only in a general sense that is not related to their particular sexual relationship (Ramathuba 2012). AIDS and the STI epidemic, especially in South Africa and sub-Saharan African countries, pose a huge challenge and have reached an alarming proportion (Lawoyin & Kanthula 2010). Hence, sexual communication between men and women in any type of intimate relationship should be viewed from a risk perspective.

Women are always vulnerable and often only get to know about their seropositive status when they become pregnant. Based on antenatal clinic surveillance, South Africa’s HIV epidemic shows no evidence of decline. There were 5.5 million adults living with HIV in 2005 – 5.3 million were adults over the age of 15 years, of whom 3.1 million were women (Friedman, Mthembu & Bam 2006). Studies have been documented on the difficulties that men and women perceive in raising the topic of infidelity and condom use within marriage (Akwara, Madise & Hinde 2003; Zulu & Chepngeno 2003). Practising safe sex involves a complicated process of sexual negotiation which requires an element of open communication about sexual issues. How sex is negotiated depends on the construction of risks and trust, both of which differ according to the type of sexual relationship or sexual encounter, as well as the inequality of the gender relations.

### Problem statement

HBC programmes in South Africa are developed inadequately compared with those of other countries around the world, because they are community-based but not community-oriented (Akintola 2004). HBC programmes in Vhembe are still in their infancy and are not properly managed; however, there are others that are formalised and have support from non-governmental organisations, offering programmes that are sustainable and can provide a career ladder for the HBCGs. They provide training in short courses that are intense and have unit standards that place the carers at a level where they are able to perform their activities exceptionally well. However these HBCGs are taken for granted as if they are just unknowledgeable volunteers and do not get the support of their communities. Moreover, there is also poor coordination between HBC, primary healthcare (PHC) and the hospitals because they are not utilised appropriately, even though they are in the forefront of health promotion. The scourge of HIV is on the increase in Vhembe as more PLWHA are discharged back to the PHC services. HBCGs do follow-up support visits of the clients to provide different services like supervising the administration of medicines, assisting in nutrition and hygiene and health promotion. However, HBCGs find it difficult to promote safe sexual practices, encourage sexual communication due to the patriarchal nature and cultural conservatism they are wrongly identified and at some instances they are no longer allowed visiting their homes as they are associated with HIV. When the community sees them visiting a household they start gossiping even when they assist with any chronic disease. It is therefore necessary that they be empowered and PHC support their activities and educate communities that they are part of the PHC team.

#### Research objectives

To explore and describe HBCGs’ perceptions of health promotion with regard to sexual health communication.

## Research design

The study explored and described perceptions of HBCGs regarding health promotion as it relates to sexual health communication (Creswell 2009). The population comprised six HIV and AIDS HBCGs who had undergone training through Tendazwau Training in Vhembe district. Vhembe district is one of the five district of Limpopo Province and has four municipalities, namely, Mutale, Makhado, Thulamela and Musina. It is a rural district having a low socioeconomic status, with the majority of its population benefiting from social grants. The district also has a high prevalence of teenage pregnancy and PLWHA. The main ethnic groups found in Vhembe are VhaVenda and Vatsongas, who still have some hold of their tradition and cultural beliefs pertaining to marriage, procreation and childrearing. Home-based carers have developed in the rural villages of Vhembe as a way of increasing accessibility to PHC and providing basic health support to communities regarding health promotion. Participants were sampled purposively as they had been working for more than a year with home-based patients in their communities and were undergoing training at the time. As CHWs, they were able to share their experiences whilst doing HIV community work and were also members of the same ethnic group. The sample consisted of three HBC groups which were selected randomly.

Five HBCGs were men, 25 were women and only three were single whilst the rest were married. Data were collected through face-to-face individual interviews with five participants who were not part of the two focus group discussions. Permission was sought from HBC centres and the participants agreed to participate in the study upon full explanation of the purpose of the research and that interviews would be audio-recorded. The central question that directed the research study during the sessions was: ‘Can you please explain your perception regarding health promotion on communication relating to sexual health amongst the clients you engage with on daily basis’. The question was further paraphrased in Tshivenda so that the informants could understand and participate effectively in their own language. Interview techniques such as paraphrasing to enhance meaning and probing to persuade the participant to give more information were used (De Vos *et al.* 2011). Interview transcripts were analysed using open coding in which data were organised into one theme and five subcategories (Creswell 2009).

## Trustworthiness

Trustworthiness followed Lincoln and Guba’s four aspects of measuring trustworthiness, namely, credibility, transferability, dependability and confirmability (Lincoln & Guba 1985). Perceptions regarding health promotion concerning sexual health communication amongst HIV home-based carers in Vhembe district were explored by being immersed in the context of HBCGs, conducting interviews and observing for 45 minutes to one hour with each participant in order to ensure credibility. The interviews were conducted at the different HBC centres at the various villages; the different settings provided information-rich data based on the context of each individual participant, thus ensuring dependability. The data that was transcribed from the interviews were not edited, were coded with co-researchers to ensure dependability. The data were confirmable as a product of the narratives of the HBCGs.

## Ethical considerations

The following ethical measures were considered throughout the process of the research in order to protect the rights of the participants (Creswell 2009; LoBiondo-Wood & Haber 2002): permission was sought from home-based organisations and participants; participants were informed that participation in the study was voluntary and that they should feel free to withdraw from the study at any time they felt uncomfortable; and codes were used instead of names in order to ensure the anonymity of the participants and to assure them that the information provided would not be used against them.

## Results and discussion

According to De Vos *et al.* (2011), concepts that seemed to pertain the same phenomenon are given a conceptual name and categories are developed in terms of their properties and dimensions. Creswell (2009) stipulates open coding as being the part of analysis that pertains to the naming and categorising of phenomena through examination of data as outlined below. Data from this study were grouped into one theme and five subthemes.

### Theme 1: Participants expressed their perceptions of socio-cultural factors affecting sexual communication

The study explored HBCGs’ perceptions of health promotion on sexual health and data from interviews and focus group discussions were arranged into a theme and subthemes. The theme was conceptualised from the narratives; and open coding of analysis led to the formulation of subthemes, as illustrated in Table 1. Socio-cultural factors were seen to impact on health promotion pertaining to sexual health communication and a description of the subthemes and literature control follows hereunder.

**TABLE 1:** Research findings.

Themes	Subthemes
Factors affecting sexual communication.	Male chauvinism.
		Misinformation about male circumcision.
		Women in any form of marital union.
		Socioeconomic status.
		Experiences of guilt feeling and blame.

#### Male chauvinism and sexual health

Communication taking place amongst partners often contributes negatively to sexual health as men usually boast about sex, feeling that having many sexual partners makes them popular and important in the eyes of their peers. One participant indicated:

‘You know, sexually-transmitted infections won’t be easily controlled as men think that it is an honour to be having more than one women; and these make every man to conform to this ill behaviour.’ (P3, female, 28 years)

Rivers and Aggleton (1999) also support the fact that men are often stigmatised if they cannot demonstrate having a wide sexual experience. In most situations, sexual decision making is usually controlled by men; and coercive sex and sexual violence are common. Another participant indicated:

‘The problem is if you tell your partner that you don’t need sex or feel like having sex, he insist [*sic*] and you are bound to give in because he starts to being angry, so sometimes you don’t have a choice; they fail to understand.’ (P5, female, 35 years)

Furthermore, another male participant said:

‘These women can surprise you, they just say they don’t need to have sex but their actions doesn’t say that, when you start brushing their thighs and fondling them, why don’t they remove your hands to show that they don’t really need it.’ (P1, male, 27 years)

Vasconceles, Garcia and Mendonca (1997), as cited by Rivers and Aggleton (1999), also found that girls and women are often forced into sex and often obey their boyfriends’ wishes because they believe that women are meant to be compliant and subservient.

Communication regarding healthy sexual practice is often not occurring – the participants are HIV home-based carers, but because of cultural norms within the VhaVenda, it is difficult to change the social norm. A male participant said:

‘Condoms are rarely used and opening communication about condom use among partners is a risky subject, which suggests that the other partner may be cheating; and if it’s the women who browse the subject, the man thinks the woman is trying to be smart [*where did you learn that?*] and the man will try to show the women that he is in control.’ (P2, male, 30 years)

Mulaudzi (2007) also indicated that men were not interested in using condoms, indicating that they limit sexual satisfaction. At times, ethnic minorities are resistant to changes in behaviour under pretence of culture and it is this cultural inertia that places women at risk. Ramathuba (2012) suggests that changing the traditional views of masculinity and femininity is essential in promoting sexual health amongst partners, as poor sexuality standards and habits contribute significantly to high-risk sexual behaviour. The *Constitution of South Africa Act* (No. 108 of 1996) declares equal rights for all citizens and states that women have reproductive rights and the rights to negotiate when and how to have sex; women should not be forced into having unsafe sex because they are viewed as inferior, either by men or society (South Africa 1996). Home-based carers should be empowered with basic human rights and the ability to inform communities about their sexual rights, namely, the freedom to choose to have safe sex.

#### Misinformation about male circumcision and sexual health communication

Another perception of risk behaviour is the traditional practice of male circumcision as it influences sexual behaviour. Men who have been circumcised think that they are less prone to infection or transmission of disease, often boasting about it. One participant said:

While talking with other men, encouraging them to use condoms, they laugh and tell you that they have been circumcised (*ro pfumba muthannga*). (P7, male, 32 years)

Men resist the use of condoms, thinking that once one is circumcised, there is no risk of being infected. Furthermore, the participant indicated that:

‘[*t*]he majority of men indicated that they were not using a condom as it reduced sexual pleasure and that they did not belief [*sic*] that their primary partners can place them at risk.’ (P7, male, 32 years)

One female participant also indicated fear of initiating condom use with her partner since it is seen as a sign of mistrust in a relationship. She said:

‘If I ask my husband to use a condom, he will scold me and ask me whether I still trust him or not.’ (P10, female, 24 years)

These statements reflect a lack of sexual knowledge with regard to the fact that even primary partners are at risk of contracting STIs – condom use is associated with a partner’s confession of infidelity, rather than being seen as a method of prevention. Health promotion should be intensified so that men can see the value of condom use; and HBCGs, as health promoters, should assist in the campaigns to reinforce condom use amongst the communities they serve and to change the behaviours and attitudes of the community members. According to the South African survey report by the Human Sciences Research Council, released in April 2014 (Khan 2014), it was found that condom use is on the decline and the Minister of Health has made a plea for consistent condom use, whether circumcised or not, although the Minister encourages medical circumcision, as it reduces (but does not prevent) the risk of transmission of STIs. Home-based carers should dispel the myths and perceptions regarding circumcision being seen as a ticket to a clean bill of sexual health.

#### Women in any form of marital union and sexual health communication

Marital status influences the perception of the risk of HIV infection and sexual behaviour – there is often a perception that married women are unlikely to have STIs as compared to unmarried women. However, Lear (1995) indicates that those in casual relationships only use condoms until or unless the relationship becomes more committed. Where sexual encounters occurred between heterosexual friends, condoms were less likely to be used because the partner was trusted by virtue of being a friend. One participant said:

‘I observed my husband undressing and was messy around the penile area with pieces of toilet papers adherent around the pubic area, but it was so difficult for me to confront the issue, you just beat about the bush and generalise the subject to say people will die; it’s dangerous outside as HIV/AIDS is all over.’ (P8, female, 30 years)

Relationships should be constructed around trust, but most men live under the pretence that they are faithful to their partners whilst exposing them to STIs. Lear (1995) indicates that:

[*w*]hen a person learns that a partner has been unsafe with someone else, it causes a re-evaluation of what would be required to trust in the future. These are the type of traumatic incidents that may cause people to change their ideas about trust, their attitudes and practices.

Zulu and Chepngeno (2003) also reported in their study that married people express their fears and anxieties to their partner because they recognise that controlling their own behaviour is insufficient. Discussion about HIV risk preventive strategies within marriage is often introduced with reference to specific stories and events relating to the infection; these discussions are conducted cautiously so as to avoid marital conflicts and disagreement. This indicates how culture impacts on the female gender as women are required to be submissive. Akwara *et al.* (2003) reported that married women face extra challenges as ‘most women (63%) perceived their risk of HIV infection to be moderate or great, with the reason being that they suspected that their spouse/partner had other sexual partners’. Whilst 46% of men perceived themselves as being at moderate to high risk because they had many sexual partners, fewer than one in five perceived themselves to be at high risk because of not using condoms. This results in women being fearful of their partner’s risky behaviour. Another participant said:

‘There are times where you suspect that your partner might be seeing someone.’ (P9, female, 25 years)

Double standards of sexual morality place women at risk because society assumes that male sexuality is biologically uncontrollable and hence inevitable, despite the fact that in some ethnographic studies in Nigeria, where:

society confers on women some right to refuse sex to an infected husband, this right does not extend to refusal on the ground of philandering. This has a great implication for the transmission of HIV/AIDS. (Ogunjuyigbe & Adeyemi 2005)

Varga (1997) also states that that power inequity and emotional and financial dependency present significant obstacles to sexual decision making, as women are unable to exert an influence over condom use because of a fear of rejection and stigmatisation by their partners. This leads to sexual risk taking in the form of unprotected sex as a means of maintaining both social and economic survival. Home-based carers as community members are aware of the different ethnic groups in the communities and some of the practices. Because of this, they should be able to align their health promotion based on the context, so as to address the social ills that prevail in the communities. Women should be seen as human beings who have human rights, who deserve to be treated with dignity and have reproductive rights, even if they are in a polygamous marriage.

#### Socioeconomic status and sexual health communication

Generally disclosure of a seropositive status is stressful and can cause strain and feelings of insecurity and mistrust in an intimate relationship. Desgrées du Loû (2005) reported that women who had not informed their partners about their status had been motivated by fear that they would be abandoned, leaving them destitute. Women prefer to remain silent because of the social and emotional stigmatisation associated with a seropositive status.

One of the participants stated:

‘One woman found out that she is infected and it is difficult to disclose her status to his partner, she found out when giving birth to her child, but she still continues to breastfeed her child whilst knowing the implications of her actions since her husband will suspect her if she does not breastfeed, moreover she is not working.’ (P15, female, 26 years)

Women lack the ability to negotiate and communicate their needs and aspirations in a sexual relation because of patriarchal and social orientation. This leads to them dying silently as a result of sexually-related illnesses because men have almost total power and control over women in this setting. Women have less negotiating skills and cannot negotiate condom use or tell their husbands to get tested because condom use within marriage has a negative image and is associated with promiscuity. Negotiating condom use can be a sensitive subject. Ramathuba (2012) also reiterates that condoms are more associated with extramarital affairs and infidelity, so bringing up the subject for discussion might lead to feelings of mistrust. According to Muhwava (2004), condom use in marital settings is unlikely to change unless social norms change, because [*in Zimbabwean culture*] powerful norms surrounding the concept of masculinity discourage condom use in marital relationships. Furthermore, the participant indicated that:

‘[*t*]he woman indicated that if she can disclose, she fears being rejected or the husband leaving him as she has no other form of support.’ (P15, female, 26 years)

Poverty or economic dependency also places women at risk as women feels helpless and hopeless once they come to terms with the knowledge of their status. Because of the fear of rejection and lack of social support, they resort to keeping quiet for the sake of support and of maintaining relationships. Unfortunately, women often maintain a relationship at the expense of their health concerns, thus making them unable to deal with concerns related to safer sex. Home-based carers should be able to change the perceptions of women in order to free them from the bondages of poverty. Merely offering health education is not enough; they should encourage women to start with self-help projects, so as to be economically sound. When women engage themselves in some form of social support, they become empowered and emancipate themselves and are then able to assist others.

#### Experiences of guilt resulting from multiple partners

Women faces humiliation and disrespect every time there is a sexual health problem in their relationship and the blame is placed upon them. This stereotype is common amongst the VhaVenda in that when a person has an STI, they say he has ‘*malwadze a vhasadi*’ – a woman’s disease. One participant asked:

‘What is the use of discussing the illness with your husband knowing that he would not succumb to the truth or accept to be treated?’ (P4, female, 31 years)

Another said:

‘There is no need to talk about it; it’s your sexual health problem with all the blame placed on you, whilst knowing that you have been faithful all along.’ (P17, female, 28 years)

This type of situation is common amongst families in that the women are blamed, even when they are innocent and not partaking in sexual relations – men are the ones with multiple partners and are tolerated, even when they bring the infection back to their partners. Mulaudzi (2007) also reported this common belief amongst various cultures, in that these are diseases transmitted by women; however, polygamy is accepted in most cultures or at least having a mistress in a relationship is acceptable, which is a problem that perpetuates the spread of STIs. Blame shifting is always the norm when communication is not happening amongst partners. Women often die in silence, but if partners and couples were empowered regarding how to open up lines of communication and be frank, we would not be experiencing the perpetuation of HIV in this day and age. Home-based carers as community counsellors and health promoters have to encourage communities to be open, truthful and frank about their HIV status to their loved ones and families in order to receive support. They also need to identify problem areas and refer the individuals to the PHC facilities for further counselling and support.

## Recommendations for promotion of communication relating to sexual health

The findings of the study revealed that communication about sexuality amongst men and women is not frank and that this can lead to communication difficulties in relationships. Communication barriers occur as a result of certain stereotyped behaviours and this can also hamper health-seeking behaviour. With this in mind, the following recommendations were made:

- Health education and awareness campaigns should focus on changing the perceptions and addressing the socio-cultural determinants affecting men and women, specifically with regard to their protection from HIV and other STIs.
- Sexuality, reproductive health and rights should be reinforced so as to build a culture of respect and equality in relation to sexual issues and protecting each other from HIV and STIs.
- Men should also be included in reproductive health counselling; they should be counselled on the consistent and correct use of condoms and the importance of remaining faithful to their partners.
- Partners in any type of sexual relations should be empowered to communicate freely and have control over matters related to their sexuality.
- Primary healthcare should derive a health promotion model in collaboration with HBCGs in order to strengthen the delivery of the services.
- Providers of healthcare should strengthen the linkages and collaborations with HBCGs as an inclusive part of the multidisciplinary team and provide them with programmes that will empower them in their role as health promoters.
- Partners should be empowered to gain skills in negotiating safer sex; and providers should encourage partner communication.

## Limitations of the study

The study was conducted in one district of Limpopo Province with a limited sample of only three HBC groups. Generalisation of the findings is thus not possible.

## Conclusion

The study explored home-based carers’ perceptions regarding health promotion about communication relating to sexual health. Communication seemed to be lacking or not taking place at all, which is an implication for STIs and HIV. Home-based workers at grass-roots level within communities are in a better position to intensify health promotion activities and/or programmes, thus easing the workload burden of nurses in the public hospitals because they are not able to intensify health education programmes. Men need to be co-opted into reproductive health programmes so that they can protect their own health. Women should be empowered to be able to negotiate safe sex practices and to be able to communicate their aspirations and expectations in a frank and assertive manner. Empowering HBCGs with the necessary skills and knowledge may assist in reducing the scourge and expel the myths and stereotypes related to sexual health, as well as improving sexual health communication.
